# Depressive Symptoms Among Higher Education Students in Germany—A Systematic Review and Meta-Analysis

**DOI:** 10.3389/phrs.2024.1606983

**Published:** 2024-06-10

**Authors:** Eileen Heumann, Ana Valentina Palacio Siebe, Christiane Stock, Katherina Heinrichs

**Affiliations:** ^1^ Institute of Health and Nursing Science, Charité—Universitätsmedizin Berlin, Corporate Member of Freie Universität Berlin and Humboldt-Universität zu Berlin, Berlin, Germany; ^2^ Unit for Health Promotion Research, University of Southern Denmark, Esbjerg, Denmark

**Keywords:** depression symptoms, higher education students, mental health, meta-analysis, prevalence

## Abstract

**Objectives:**

Higher education students in Germany are vulnerable to depressive symptoms. Despite ample evidence, no comprehensive review has recently been conducted. Thus, our systematic review and meta-analysis aims at describing the extent to which students are affected by depressive symptoms.

**Methods:**

We searched three databases for articles reporting the prevalence rates of depressiveness among students in Germany published between 2002 and 2023. Pooled prevalence rates were calculated using random effects models, both for the overall sample and for subgroups categorized by gender, study setting, assessment instrument, and whether the study was conducted before or during the COVID-19 pandemic.

**Results:**

The search yielded 992 records. After screening, 60 articles remained for data extraction. About one out of five students (21.1%) exhibits depressive symptoms. Pooled prevalence rates differ between subgroups, with higher rates during the COVID-19 pandemic than before (30.6% versus 18.0%) and with females being more affected than their male counterparts (29.0% versus 23.1%).

**Conclusion:**

This review underlines the urgency with which the mental health of students should be addressed at the (higher educational) policy level.

**Clinical Trial Registration:** PROPSPERO, Identifier CRD42022384066.

## Introduction

When entering higher education, students are exposed to a number of new and stressful experiences that might affect their mental health [1, 2]. The pressure for academic success, competitive environments within study programs, concerns about post-graduation prospects as well as moving away from home, taking on financial responsibilities for the first time and finding an accommodation are likely to contribute to higher education students’ severe stress, anxiety, and depressive symptoms [2, 3].

Previous international systematic reviews show that prevalence rates of depressive symptoms in higher education students and certain subgroups are generally high [4–6]. According to these findings, more than one-quarter of higher education students suffer from depressive symptoms [4]. In Germany, cross-sectional studies reveal similarly high levels of depressive symptoms [7–9], and compared to the general population, higher education students are more likely to develop depressive symptoms [10].

Some groups of higher education students show higher rates of depressive symptoms [5]. For example, female students [11] and individuals with low socio-economic status [6] are considered to be more at high risk of developing depressive disorders. Similarly, sexual and gender minorities commonly exhibit more depressive symptoms [12]. Furthermore, students who possess limited social support resources [13], face interpersonal stressors (e.g., in romantic and peer relationships) [11], have pre-existing health conditions, or have experienced traumatic events or childhood adversities [5] seem to have a higher prevalence of depressive symptoms.

Evidence suggests that psychological problems in higher education students are associated with comprised academic performance and diminished productivity [14, 15] as well as an elevated risk of discontinuing studies [16]. Apart from this, depressive symptoms in higher education students are associated with risk behaviors like illicit substance use or frequent binge drinking [17, 18] as well as with lower levels of physical activity [19]. Moreover, higher education students who report depressive symptoms are more prone to self-injurious behavior and suicide ideation [20]. In conclusion, the proliferation of depressive symptoms has various negative and detrimental consequences for the affected students, the universities, and the society at large [16, 21].

Research suggests that the Coronavirus Disease 2019 (COVID-19) pandemic exacerbated the mental burden experienced by higher education students: several cross-sectional studies conducted during this period reported elevated levels of depressive symptoms among students compared to pre-pandemic times in the general population [22, 23]. Additionally, longitudinal studies [24] and cross-sectional comparisons [25] provide further evidence of the escalating levels of depressive symptoms over the course of the pandemic. The unprecedented circumstances, such as sudden shifts to online learning, reduced social interactions, financial constraints, and uncertainty about the future, have all contributed to the worsening mental health outcomes among higher education students during this challenging time period [24, 26].

Over the last decade to 15 years, there has been a growing emphasis on public mental health research among higher education students, particularly focusing on monitoring the prevalence of depressive symptoms and assessing the associated factors [4, 27, 28]. Notably, during the COVID-19 pandemic, there has been an upsurge in the number of studies conducted, especially cross-sectional studies, aimed at expanding the existing body of evidence [25, 29].

Nevertheless, no comprehensive review compiling the results of the various studies on the prevalence of depressive symptoms among higher education students in Germany is known to us. Thus, an overview on the results of previous studies is needed. The results of such a review can be used to develop certain programs in the university context to promote higher education students’ mental health. Ultimately, this research can contribute to enhancing the support systems and services available to students, aiding in the early identification and management of mental health issues, and fostering a conducive learning environment for the academic success and holistic development of higher education students.

Hence, the objective of this systematic review is to determine the extent to which higher education students are affected by depressive symptoms in Germany by addressing the following research question: What is the prevalence of depressive symptoms among higher education students in Germany? Furthermore, our objective is to ascertain the prevalence of depressive symptoms in specific subgroups (e.g., female and male students) based upon available data.

## Methods

### Literature Search Strategy and Data Sources

Our systematic review was *a priori* registered on PROSPERO (CRD42022384066). During the whole research process, we considered the preferred reporting items for systematic reviews and meta-analyses (PRISMA) [30].

The systematic literature search included three core concepts, namely, “depressive symptoms,” “higher education students,” and “Germany.” Within each concept, we used different free text terms with truncations and controlled vocabulary terms with automatic explosion, which were joined with ‘OR’. The concepts were linked using ‘AND’. Both German and English search terms were included (Table 1). We restricted our search to “Title and Abstract” in order to be more precise and to avoid obtaining results where a higher education institution is indicated in the authors’ affiliations. Moreover, filters on English or German language, peer reviewed journal, and publication date within the years 2002–2023 were applied as we decided to focus on the period after the implementation of the Bologna reform in Germany to make the results more comparable.

**TABLE 1:** Components of the search string (Depressive symptoms among higher education students in Germany–a systematic review and meta-analysis, Berlin, Germany, 2022–2023).

Core concepts	Depressive symptoms	Students	Germany
Search terms	Depress* MD* “Affective disorder*” “Mood disorder*” Affektstörung* Affektiv* Depression [MeSH] Depressive disorder [MeSH] Depressive disorder, major [MeSH]	Student* “University student” College Higher education Undergraduate Graduate Bachelor* Master* “State examination” Studierende* Staatsexamen Magister*	German* Deutsch*

MD, major depression; MeSH, medical subject headings.

We searched the databases MEDLINE and Embase through Ovid and PsycINFO through EbscoHOST, respectively for eligible publications on July 15, 2022. We set an alert, which continued until data extraction was completed on June 30, 2023. Additional searches were conducted by one author (EH) via Elicit [31] on July 26, 2023 and by reviewing the references of the identified articles and the citing literature via Google Scholar. The search in Elicit was based on the underlying research question of the review.

### Study Eligibility Criteria

We included articles which met the following criteria: 1) the population studied are students from universities or universities of applied sciences, 2) data was assessed at higher education institutions in Germany, and 3) information on depressive symptoms as outcome is provided, including diagnosis of affective disorders (International Statistical Classification of Diseases and Related Health Problems (ICD) codes F30-F39) or prevalence rates indicated by cut-off values of instruments for assessing depressive symptoms. In addition, we considered only 4) original articles published in peer-reviewed journals in 5) German or English language 6) between 2002 and 2023. Our inclusion criteria were modified during the screening process because the data appeared too comprehensive. Therefore, we decided to include only prevalence rates and not to include studies that reported mean values.

Qualitative studies, opinion papers and commentaries, review articles, and studies published only as abstracts were excluded. In addition, studies that examined depressiveness as symptoms of other conditions than affective disorders (e.g., postnatal depression) or in other population groups (e.g., high-school students) were also excluded.

### Study Selection and Data Extraction

Rayyan [32], an online tool for conducting collaborative reviews, was used to maintain the search results. Duplicates were automatically removed by the search portals and can therefore not be reported. Two reviewers (EH and KH) independently screened all results of the literature search and excluded irrelevant articles based on titles and abstracts. Cohen’s kappa was calculated for this selection step to account for interrater agreement. Full text copies were obtained and independently assessed by two reviewers (EH and KH). Publications were excluded only if the two reviewers agreed, and any disagreements were resolved by discussion until consensus was reached. Reasons for exclusion were documented during full-text screening.

One reviewer (EH) extracted data from all included publications using a standardized excel spreadsheet. The following information was extracted and synthesized: bibliographical data (first author’s name, year of publication), study design, period of data assessment, details on study population (sample size, higher education institution, age and gender distribution, study field(s)), instruments used to assess depressive symptoms, prevalence of depressive symptoms in the whole sample and additional prevalence rates (e.g., for subgroups)—if applicable.

### Quality Appraisal

The appraisal of study quality was conducted by two authors, EH and KH, ensuring a thorough and objective assessment process during which each person checked the other person’s appraisal. By employing the Joanne Briggs Institute (JBI) Critical Appraisal Tool for Studies Reporting Prevalence Data [33], we ensured a rigorous and standardized assessment of the quality of prevalence studies included in our review. This tool allowed us to systematically assess various aspects of the studies, enhancing the credibility and reliability of our findings. The JBI Critical Appraisal Tool is commonly utilized for evaluating the quality of prevalence studies [34, 35], and its use has been recently recommended following a systematic review of quality assessment tools [36]. This checklist comprises 9 items, which assess various aspects of the study, including the representativeness of the sample, appropriateness of recruitment methods, adequacy of sample size, detailed description of subjects and setting, valid ascertainment and measurement of the condition, thorough reporting of statistical analysis, use of standard measurements for all participants, and adequacy of the response rate. Although the tool does not provide a numerical score, we assigned proportions to rate the quality of the included articles. However, poor-quality articles with a high risk of bias were not excluded from data synthesis as this could lead to selection bias [37] and their contributions will be thoroughly discussed within the context of the overall discussion.

### Data Synthesis and Statistical Analysis

For descriptive data synthesis, a comprehensive table was generated, presenting the sociodemographic characteristics of each study along with their respective prevalence rates of depressive symptoms among higher education students. This tabular format provides an overview over the individual study findings and their respective sample compositions.

Additionally, meta-analyses were undertaken using SPSS Version 29.0 to synthesize the data. Given the variability in prevalence and variance within studies and as recommended by Borges Migliavaca et al. [38], a random effects model was employed. The range of effects was evaluated through visual examination of the forest plot depicting estimates and 95% confidence intervals (CIs), along with consideration of the weight assigned to each point estimate. For studies in which more than one subgroup prevalence rate was reported for the entire sample, a weighted mean was calculated to avoid distorting the *p*-value of the meta-analysis by including multiple data points per study.

Subgroup analyses were carried out to explore heterogeneity among studies. The subgroups were defined based on sex (male/female), field of study (medicine and dentistry), first-year students only, and time-point of data collection (before/during the COVID-19 pandemic). Subgroup analyses were also conducted for the two most frequently used screening tools, the Patient Health Questionnaire (PHQ) and the Beck Depression Inventory (BDI). Differences between subgroups were assessed based on 95% CIs. Moreover, the I^2^ statistic was utilized to assess the extent of study heterogeneity. Values above 25%, 50%, and 75% were considered to represent low, moderate, and high levels of heterogeneity, respectively [39]. A funnel plot was additionally created to visualize the potential heterogeneity and publication bias.

In case of sample overlap across multiple articles, for example, by utilizing the same dataset, only the one with the largest sample size was incorporated into the meta-analysis. This was done to mitigate potential bias. If studies used multiple assessment instruments, we included results from the instrument validated and established in student mental health research and if more than one prevalence rate was reported (e.g., for sex-specific subgroups only), we calculated a weighted mean for consideration in the meta-analysis.

In case of longitudinal studies with multiple time points, our meta-analysis focused on using the baseline prevalence data, as it was done in previous student mental health research, e.g., by Ahmed et al. [40]. This decision was primarily based on two considerations. Firstly, the baseline data frequently represents the largest sample size among all data collection time points, ensuring a more robust estimate of prevalence. Secondly, using baseline data helps to minimize potential confounding factors, such as demand characteristics or selection bias, which might arise at subsequent data collection time-points due to biased study participation and external factors occurring over time [40]. In addition, the inclusion of data from interventional studies for mental health would also result in selection bias.

## Results

### Literature Search

**FIGURE 1:**
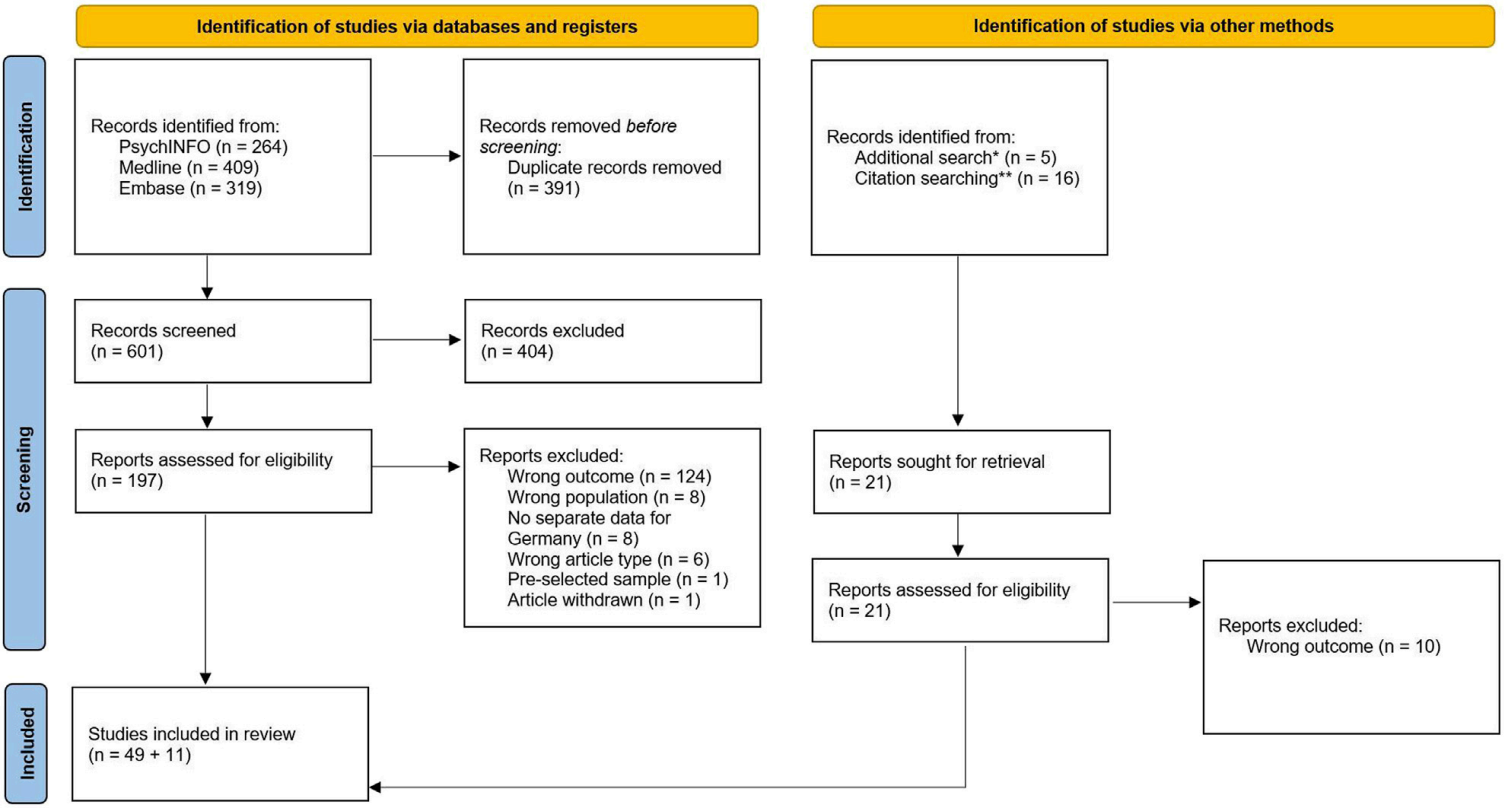
PRISMA flow diagram [44] for literature search on depressive symptoms among higher education students in Germany (Depressive symptoms among higher education students in Germany—a systematic review and meta-analysis, Berlin, Germany, 2022–2023).

Figure 1 shows the literature selection process. The initial search resulted in 911 records, and the ongoing alert yielded a further 81 records. We retrieved another 16 records through cross-checking the references and citing literature as well as through an additional search via Elicit (5 articles). After removing duplicates, 622 articles remained for title and abstract screening, of which 38 were inconsistently judged by the two reviewers (EH and KH). The resulting Cohen’s kappa was κ = 0.9, indicating a level of almost perfect agreement (93.9%). Of the 622 articles, 404 were excluded because they did not meet the eligibility criteria, leaving 218 articles for full text analysis. After full text screening, 60 articles remained for data extraction, and 56 could be used for meta-analysis. A total of four articles were excluded from the overall meta-analysis due to sample overlap as mentioned above. These articles comprised Berger et al. [41], Burger et al. [42], Mikolajczyk et al. [43], and Weber et al. [7].

### Characteristics of the Included Studies

Table 2 provides a comprehensive overview of the study characteristics found within the included articles. Most studies employed a cross-sectional design (n = 51). The sample sizes varied, ranging from n = 50 [45] to n = 7,203 participants [46], with a total of 75,107 participants and a mean sample size of 1,252 persons per study. The articles were published between 2008 and 2023, and data were gathered from 2004 to 2022.

**TABLE 2:** Characteristics of included studies (Depressive symptoms among higher education students in Germany–a systematic review and meta-analysis, Berlin, Germany, 2022–2023).

First author (citation)	Study population						Instruments	Outcome		Quality appraisal
	Sample size (n)	Institution	Age in years: Mean (SD)	Sex: Female (%)	Study fields	Year of course (year)		Cut-off score	Prevalence rate (%)	
Akhtar et al. [48]	122	n. r.	24.9 (3.6)	62.0	Medicine	n. r.	Major Depression Inventory (MDI)	≥25	26.0	5/9
Bailer, Schwarz [28]	1,130	University of Mannheim	22.8 (2.8)	55.4	Mixed (>5)	n. r.	Patient Health Questionnaire (PHQ-9)	≥10	6.0	7/9
Bastemeyer et al. [49]	648	n. r.	22.6 (3.3)	36.1	Sports	BA: 1.-2., 5.-6. MA: all	Patient Health Questionnaire (PHQ-2)	≥3	11.6	4/9
Berger, Franke [41]	293	University of Heidelberg	22.9 (3.7)	73.0	Medicine Psychology	n. r.	Psychosocial complaints list (PCL)	≥3	6.5	7/9
Burger, Tektas [50]	530	n. r.	21.9 (3.4)*	55.5*	Medicine	1.-2.	Beck Depression Inventory (BDI-II)	≥14	12.1^a^	5/9
Burger, Neumann [42]	163	Friedrich Alexander University of Erlangen-Nürnberg	1. sem.: 21.3 (n. r.) 2. sem.: 21.4 (n. r.) 4. sem.: 22.7 (n. r.) 5. sem.: 23.3 (n. r.)	1. sem.: 68.7 2. sem.: 70.2 4. sem.: 60.0 5. sem.: 74.2	Dentistry	1.-3.	Beck Depression Inventory (BDI-II)	>9	1. sem.: 40.5 2. sem.: 46.5 4. sem.: 51.2 5. sem.: 78.1	5/9
Burger et al. [51]	758	Friedrich Alexander University of Erlangen-Nürnberg	n. r.	57.8	Medicine, Dentistry, Molecular Medicine	1.-3.	Beck Depression Inventory (BDI-II)	≥14	16.9^a^	6/9
Chow et al. [52]	251	Heinrich Heine University Düsseldorf	22.2 (4.7)	71.3	Medicine	2.	Patient Health Questionnaire (PHQ-8)	≥10	20.7	6/9
Ehring et al. [53]	1,108	Martin-Luther University Halle-Wittenberg	23.1 (3.9)	65.0	Medicine	1.-3.	Beck Depression Inventory (BDI-II)	≥14	19.0	9/9
Eissler et al. [54]	4,095	Ulm University (UU), Ulm University of Applied Sciences (HU), Neu-Ulm University of Applied Sciences (HNU), Cooperative State University Baden-Württemberg (DHBW)	UU, HU, HNU: 23.1–23.4 (n. r.), DHBW: 27.2 (n. r.)	53.7*	n. r.	n. r.	Patient Health Questionnaire (PHQ-2)	≥3	20.3^a^	6/9
Feussner et al. [55]	271	Martin-Luther-University Halle-Wittenberg	23.5 (3.8)*	68.6*	Medicine, Dentistry	Med.: 3., Dent.: 1.-5.	Beck Depression Inventory (BDI-II)	≥14	31.1^a^	5/9
Gecht et al. [56]	506	n. r.	20.8 (1.9)	30.0	Medicine, Mechanical Engineering	n. r.	Rasch-based Depression Screening (DESC)	≥12	10.5	4/9
Gräßel et al. [57]	146	n. r.	20.9 (3.7)	65.1	Medicine	1.	Hospital Anxiety and Depression Scale (HADS-D)	≥8	6.8	6/9
Guse et al. [58]	887	University Medical Center Hamburg-Eppendorf	n. r.	63.4	Medicine	1.-4.	Patient Health Questionnaire (PHQ-2)	≥3	20.6	9/9
Halfmann et al. [59]	561	Mannheim Medical Faculty of the University of Heidelberg, Würz-burg Medical Faculty	23.4 (4.0)	74.5	Medicine	n. r.	Hospital Anxiety and Depression Scale (HADS-D)	≥8	5.5	8/9
Haussleiter et al. [60]	2,329	Ruhr-University Bochum	24.3 (4.0)	64.4	n. r.	n. r.	Beck Depression Inventory (BDI-II)	≥20	19.5	7/9
Heinen et al. [61]	321	University of Hamburg	21.8 (3.9)	60.4	Medicine	1.	Patient Health Questionnaire (PHQ-2)	≥3	11.5	8/9
Heumann et al. [46]	7,203	Martin-Luther University Halle-Wittenberg, University of Bremen, University of Siegen, Charité—Universitätsmedizin Berlin, Heinrich Heine University Düsseldorf	24.1 (5.0)	67.0	Mixed (>5)	n. r.	Patient Health Questionnaire (PHQ-2)	PHQ-2 ≥ 3	PHQ-2: 28.6	7/9
Hilger-Kolb et al. [62]	689	n. r.	22.7 (2.7)	69.5	Medicine, “non-medical subjects"	n. r.	Patient Health Questionnaire (PHQ-2)	≥3	15.8	7/9
Holm-Hadulla et al. [22]	2,135	University of Heidelberg	n. r.	66.5	Mixed (>5)	n. r.	Patient Health Questionnaire (PHQ-9)	≥10	59.1	7/9
Huckert et al. [63]	137	University of Trier	22.3 (2.3)	55.5	n. r.	n. r.	Beck Depression Inventory (BDI-II)	≤13	24.4^a^	5/9
Jurkat et al. [64]	338	Justus-Liebig University Giessen	22.5 (3.5)	61.0	Medicine, Dentistry	Med.: 1., 4., Dent.: 1., 2., 4., 5.	Beck Depression Inventory (BDI-I)	≥18	4.5^a^	6/9
Jurkat et al. [65]	651	Justus Liebig University Giessen	22.1 (n. r.)	61.3	Medicine	1., 7.	Beck Depression Inventory (BDI-I)	≥11	18.9	7/9
Kappner et al. [66]	224	n. r.	22.8 (3.6)	77.2	Medicine, Dentistry	n. r.	Patient Health Questionnaire (PHQ-9)	-	28.1	5/9
Karing et al. [67]	2548	n. r.	23.7 (4.6)	74.8	n. r.	n. r.	Patient Health Questionnaire (PHQ-8)	≥10	35.9	6/9
Karing et al. [45]	50	n. r.	24.1 (6.2)	84.0	n. r.	n. r.	Patient Health Questionnaire (PHQ-8)	≥10	32.0	5/9
Kindt et al. [68]	673	n. r.	21.6 (3.7)*	69.8*	Medicine, Psychology	n. r.	Beck Depression Inventory (BDI-II)	≤13	78.0	6/9
Kohls et al. [69]	419	Ludwig-Maximilians-University of Munich, University Witten/Her-decke	n. r.	71.0*	Medicine	n. r.	Center for Epidemiological Studies Depression Scale (CES-D)	>17	25.6^a^	5/9
Kohls et al. [29]	3,382	n. r.	24.0 (4.7)	70.2	n. r.	n. r.	Patient Health Questionnaire (PHQ-9)	≥10	37.0	9/9
Kohls et al. [70]	5,474	6 universities in Saxony	23.7 (4.8)	69.0	n. r.	n. r.	Patient Health Questionnaire (PHQ-9)	≥10	35.5	7/9
Kötter et al. [71]	881	University of Lübeck	20.4 (2.7)	54.2	STEM, Medicine	1.	Hospital Anxiety and Depression Scale (HADS-D)	>8	6.1^a^	8/9
Kreß et al. [72]	125	University of Heidelberg	22.7 (2.8)	72.0	n. r.	n. r.	Psychosocial complaints list (PCL)	≥4	17.0	3/9
Mekhemar et al. [73]	211	All dental schools	n. r.	73.5	Dentistry	n. r.	Depression, Anxiety and Stress Scales (DASS-21)	≥10	19.0	6/9
Mikolajczyk et al. [43]	565	University of Bielefeld	n. r.	58.3	n. r.	n. r.	Modification of Beck Depression Inventory (M-BDI)	≥35	25.1^a^	4/9
Mikolajczyk et al. [74]	803	University of Bielefeld	n. r.	57.7	n. r.	n. r.	Modification of Beck Depression Inventory (M-BDI)	≥35	23.0	7/9
Möller-Leimkühler et al. [75]	1,020	n. r.	23.3 (3.2)	57.7	n. r.	n. r.	Shortform of Center for Epidemiological Studies Depression Scale (CES-D)Gender-Sensitive Depression Screening (GSDS)	CES-D ≥ 18 GSDS-26 ≥ 18	CES-D: 36.1 GSDS-26: 45.0	4/9
Möller-Leimkühler et al. [76]	995	Ludwig-Maximilians-University of Munich	24.1 (n. r.)	49.4	Mainly medicine, dentistry	n. r.	Gotland Scale for Male Depression	≥13	25.6	4/9
Müller et al. [9]	3,353	University of Münster	n. r.	67.0	n. r.	n. r.	Patient Health Questionnaire (PHQ-2)	≥3	31.5	7/9
Ochnik et al. [77]	270	n. r.	n. r.	71.5	n. r.	n. r.	Patient Health Questionnaire (PHQ-8)	≥10	37.8	4/9
Pelzer et al. [78]	220	Martin-Luther University Halle Wittenberg	20.5 (3.43)	70.9	Medicine	1.	Beck Depression Inventory (BDI-II)	≥14	17.3	5/9
Polujanski et al. [79]	184	2 medical schools	22.0 (3.20)	71.0	Medicine	1.	Patient Health Questionnaire (PHQ-9)	≥10	9.8	4/9
Prinz et al. [47]	182	Friedrich-Alexander-University Erlangen/Nürnberg	n. r.	59.3	Medicine, dentistry	4., 5.	Hospital Anxiety and Depression Scale (HADS-D)	≥11	2.2^a^	3/9
Pukas et al [80]	1,103	n. r.	23.1 (4.0)	64.9	Medicine	1., 3., 5.	Beck Depression Inventory (BDI-II)	≥14	19.0	9/9
Rabkow et al. [8]	306	Martin-Luther-University Halle-Wittenberg	20.9 (2.32)	52.3	Law	1.-7.	Beck Depression Inventory (BDI-II)	≥14	33.3	9/9
Schlarb et al. [81]	2,443	Christian-Albrechts-Universtiy Kiel	24.0 (3.8)	65.0	Mixed (>5)	1.-15.	Patient Health Questionnaire (PHQ-9)	≥10	14.6	9/9
Schlarb et al. [82]	2,646	University of Tübingen, University of Koblenz-Landau	23.8 (3.7)	73.7	n. r.	n. r.	Patient Health Questionnaire (PHQ-9)	≥10	25.3	8/9
Schunter et al. [83]	913	n. r.	23.6 (4.0)	90.7	Veterinary medicine	n. r.	Patient Health Questionnaire (PHQ-9)	≥10	45.9	8/9
Schwaab et al. [84]	203	University of Heidelberg	25.2 (3.7)	58.0	Medicine	3.-5. of clinical studies	Patient Health Questionnaire (PHQ-9)	≥10	12.0	8/9
Seeliger et al. [85]	298	University of Hamburg	21.3 (4.1)	63.4	Medicine	1.	Patient Health Questionnaire (PHQ-9)	≥10	3.4	7/9
Seliger et al. [86]	390	University of Leipzig	22.4 (2.5)	58.2	Medicine	n. r.	Patient Health Questionnaire (PHQ-9)	≥10	8.8	6/9
Seweryn et al. [27]	797	University of Munich, Otto von Guericke University	22.4 (2.5)*	76.8*	Technology, Medicine	n. r.	Beck Depression Inventory (BDI)	≥11	38.9^a^	7/9
Supke et al. [23]	928	>4 universities	23.6 (3.9)	63.5	Mixed (>5)	n. r.	Patient Health Questionnaire (PHQ-9)	≥10	56.4	7/9
Tsiouris et al. [87]	4,351	Johannes Gutenberg University Mainz	23.8 (4.4)	70.5	Mixed (>5)	n. r.	Patient Health Questionnaire (PHQ-9)	≥10	29.0	8/9
Velten et al. [88]	1,275	Ruhr-University Bochum	24.0 (4.7)	61.8	n. r.	n. r.	Depression, Anxiety and Stress Scales (DASS-21)	≥7	24.3	7/9
Voltmer et al. [89]	153	University of Lübeck	25.6 (3.1)	71.3	Medicine	5.	Hospital Anxiety and Depression Scale (HADS-D)	≥8	7.4	6/9
Weber, M. et al. [90]	363	>3 universities in Berlin	25.9 (4.7)	68.0	Mixed (>5)	n. r.	Patient Health Questionnaire (PHQ-8)	≥10	38.8	8/9
Weber, R. et al. [91]	4,894	University of Cologne	24.3 (4.9)	75.3	Mixed (>5)	n. r.	Patient Health Questionnaire (PHQ-9)	≥10	20.6	6/9
Weber, R. et al. [7]	4,894	University of Cologne	24.3 (4.9)	75.3	n. r.	n. r.	Patient Health Questionnaire (PHQ-8)	-	20.6	7/9
Wege et al. [92]	592	Heinrich Heine University Düsseldorf	21.1 (3.9)	70.0	Medicine	1.	Patient Health Questionnaire (PHQ-9)	≥10	10.5	9/9
Wörfel et al. [93]	1,707	n. r.	23.3 (3.7)	73.1	Mixed (>5)	1.-5.	Patient Health Questionnaire (PHQ-2)	≥3	14.2	7/9

SD, standard deviation; n. r., not reported; sem., semester; STEM, science, Technology, Engineering, Mathematics; Med., medicine; Dent., dentistry; BA, bachelor; MA, master.

^a^ self-calculated weighted mean value.

Among the studies, 29 focused on medical and/or dental students, while 15 publications reported data on sex-specific prevalence rates. Notably, 15 studies were conducted during the pandemic, and 13 studies specifically provided information regarding first-year students. A diverse range of 14 different assessment instruments were utilized to measure depressive symptoms. Most of the studies employed versions of the PHQ (PHQ-2/PHQ-8/PHQ-9) in 30 instances, followed by the BDI (BDI-I/BDI-II/M-BDI) in 16 cases and the Hospital Anxiety and Depression Scale - Depression subscale (HADS-D) in 4 instances. Different cut-off levels were applied across these assessment instruments. Studies utilizing the PHQ primarily reported at least moderate levels of depressive symptoms, while those employing the BDI mostly indicated at least mild depressive symptoms.

The prevalence rates of depressive symptoms exhibited considerable variability across the studies. In general, the rates ranged from 2.2% (weighted mean prevalence rate for the overall sample [47]) to 59.1% [22]. For female students, the rates ranged from 5.9% [28] to 59.8% [23], while for male students, the rates ranged from 6.2% [28] to 49.2% [23].

### Quality Assessment

All 60 articles were assessed based on the nine items of JBI Critical Appraisal Tool for Studies Reporting Prevalence Data [34]. The score of each included publication can be found in Table 2. The assessment of these publications resulted in cumulative scores that ranged from 3 to 9, with a mean score of 6.3, out of a possible 9 points. The majority of studies employed suitable sampling methods (e.g., data assessment in compulsory lectures) (65.0%), provided sufficient details regarding the characteristics of their study subjects and settings (e.g., information on age and sex of the participants) (66.7%), and maintained adequate sample sizes that ranged from 306 [9] to 7,203 participants [46] (68.3%). Of 60 studies, 96.7% used an appropriate sample frame to address the target population, which substantially mitigates the risk of overgeneralizing the findings: a minimum sample size of 305 was determined based on the calculation for prevalence studies [94, 95]. This calculation considered an assumed prevalence rate of 20%, drawing upon findings from prior systematic reviews on depressive symptoms in comparable population groups [4–6].

The risk of bias for outcome measurement was consistently low due to the overall use of assessment instruments with clearly defined cut-offs for depressive symptoms (98.3%). A total of 68.3% of the studies utilized a consistent measurement approach for all participants. However, we were unable to confirm this for 19 studies due to missing information, such as whether assessments were conducted with pen and paper and whether a trained instructor was present.

Nearly half of the studies (n = 27) reported the prevalence rate in terms of percentages without specifying proportions, necessitating the classification of such studies as “unclear” in this regard. The response rate for 25 studies was noted to be below 10% or not appropriately managed, or in some instances, simply not reported.

### Meta-Analyses

**FIGURE 2:**
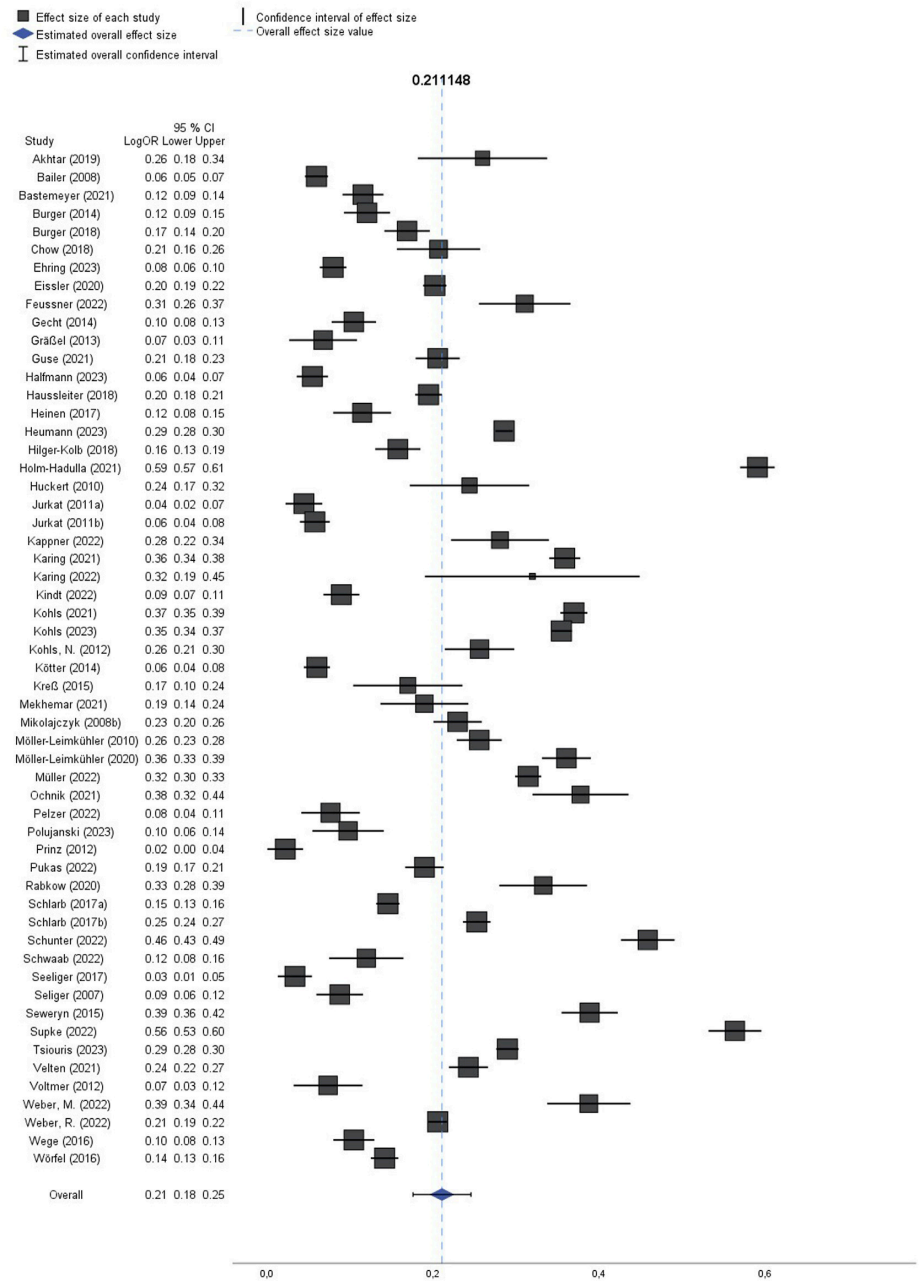
Meta-analysis of 56 studies on prevalence of depressive symptoms among higher education students in Germany (Depressive symptoms among higher education students in Germany–a systematic review and meta-analysis, Berlin, Germany, 2022–2023) * LogOR = log-transformed odds ratio.

**FIGURE 3:**
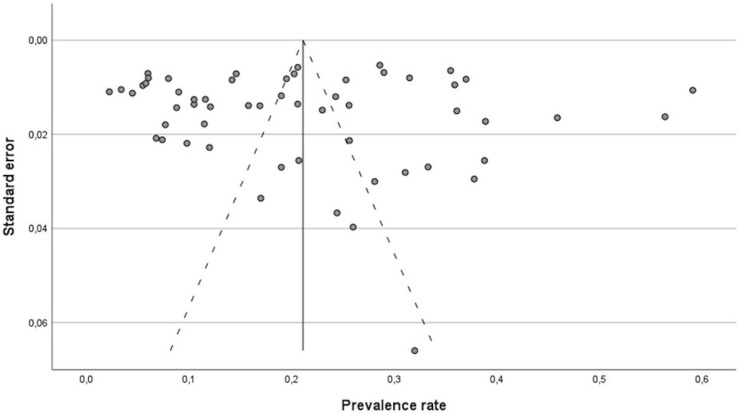
Funnel plot for publication bias (Depressive symptoms among higher education students in Germany–a systematic review and meta-analysis, Berlin, Germany, 2022–2023).

In our analysis, we found a pooled prevalence rate of 21.1% (95% CI: 17.6%–24.6%) for depressive symptoms among the higher education student population (Figure 2). The funnel plot showed no publication bias but reflected the large heterogeneity of results (Figure 3), and so did the I^2^ statistic (99%). When analyzing studies using the same assessment tool only, the pooled prevalence rates for moderate depressive symptoms based on the PHQ scale and at least mild depressive symptoms using the BDI scale were 24.8% (95% CI: 19.5%–30.1%) and 22.4% (95% CI: 17.4%–27.3%), respectively.

When examining subgroups (Supplementary File S1), we observed that the pooled prevalence rate for female students was 29.0% (95% CI: 21.4%–36.5%), while it was lower for male students at 23.1% (95% CI: 16.5%–29.6%). Furthermore, when considering the subgroups of higher education students, medicine or dental health education students exhibited a pooled prevalence rate of 13.2% (95% CI: 9.9%–16.4%). Among first-year students, the pooled prevalence rate was 11.0% (95% CI: 7.7%–14.3%), indicating a relatively lower prevalence in this subgroup. Our analysis revealed variations in the prevalence of depressive symptoms before and during the COVID-19 pandemic. Prior to the pandemic, the pooled prevalence rate was 18.0% (95% CI: 14.7%–21.2%), but during the pandemic, it was considerably higher than before (30.6%, 95% CI: 22.1%–39.1%).

Stratified by time-point of survey (Supplementary File S2, S3), around one-quarter of female higher education students and a fifth of their male counterparts showed depressive symptoms before the pandemic (25.7%, 95% CI: 18.8%–32.5% and 20.1%, 95% CI: 14.2%–26.1%, respectively). When measured with different versions of the PHQ, the pooled prevalence rate before the pandemic was 18.6% (95% CI: 13.5%–23.8%) and during the pandemic it was 33.7% (95% CI: 25.1%–42.2%). For the subgroup of medical and dental students, the time-point of the survey made no difference regarding the extent of depressive symptoms before the pandemic: 13.2%, 95% CI: 9.4%–17.0%, during the pandemic: 13.3% (95% CI: 7.6%–19.0%). During the pandemic, the BDI was not employed for data collection, only two studies examined sex-specific prevalence rates [43, 103], and only one specifically focused on first-year students [79], thus no stratification was conducted.

## Discussion

One out of five higher education students is affected by depressive symptoms. Pooled prevalence rates vary across different subgroups, and prevalence rates were higher during the COVID-19 pandemic compared to the times before the outbreak.

Our findings underpin the high level of affection among higher education students aligning with the outcomes of the World Health Organization World Mental Health Survey conducted by Auerbach et al. [96]. In their study, they reported that 20.3% of students across 21 countries acknowledged the presence of a mental health disorder, encompassing conditions such as anxiety, depressive mood disorders, behavioral issues, and substance use disorders. Notably, the high prevalence rate also appears to surpass that of the general population in Germany. According to the German Health Update/European Health Interview Survey (GEDA 2014/2015-EHIS) [97], approximately one in ten individuals in the general German population experienced depressive symptoms, with higher levels observed among women compared to men [10].

Sex differences can also be confirmed in the higher education student population: Luo et al. [98] reported significantly higher prevalence rates of depressive symptoms among female students compared with males (30.8% vs. 28.6%, *p* < 0.001) in their systematic review and meta-analysis conducted during the pandemic in China. Further evidence emphasizes the potential sex disparities in depressive symptoms [11, 99, 100]. While our analysis did not establish statistically significant sex differences, it did uncover a notable distinction between women and men, with rates of 29.0% and 23.1%, respectively.

Throughout the COVID-19 pandemic, higher education students exhibited a notably elevated overall prevalence rate of depressive symptoms [98, 101, 102]. Deng et al. [101] conducted a global systematic review and meta-analysis, revealing a pooled prevalence of depressive symptoms at 34%. Our findings parallel this trend, with a pooled prevalence rate of 30.6% during the pandemic. Volken et al. [100] also identified a gender-adjusted effect, reporting that the prevalence of depressive symptoms was higher in both Swiss female students (30.8%) and male students (24.8%) compared to the pre-pandemic Swiss population. These findings underscore the necessity for psychological support during periods of crisis. Given the ongoing political conflicts and wars, as well as the looming climate crisis, the potential for sustained mental distress cannot be disregarded [103].

Regarding subgroups of students, the prevalence rate for first-year students appears to be lower than the prevalence measured in all students. This observation is consistent with findings by Luo et al. [98], who found higher prevalence rates in postgraduates compared to undergraduates, indicating a greater susceptibility in later phases of education. In Germany, there are only few longitudinal studies focusing on the mental wellbeing of students during their academic journey. These studies suggest a deterioration in mental health among higher education students over the course of their studies [78, 104]. However, the findings are mixed, as some cross-sectional studies indicate the opposite–a high burden at the start or an improvement in mental wellbeing by the end of the study; this is observed both in Germany [80] and internationally [105]. Additional longitudinal studies featuring representative samples are needed to establish a comprehensive perspective on students’ mental health and to identify areas requiring targeted interventions. This approach could also identify possible reasons as to why the health of higher education students varies in different phases of their studies, e.g., a selective drop-out of students with poor mental health.

Half of the studies included students from medical disciplines. This tendency may arise from convenience and proximity, as many of the studies were conducted by researchers in medical or health-related fields. In their subgroup meta-analysis, a lower prevalence of depressive symptoms (13.2%) was observed compared to the overall prevalence rate. Compared with international findings German medical students seem to be less affected: Tam et al. [106] reported an overall pooled prevalence rate of 27.0% in their overview of systematic reviews on prevalence of depressive symptoms among medical students. Despite the strong focus on research within the medical field, only a limited number of studies have reported subgroup data based on the field of study. Future research should broaden its scope to include students from various faculties and subjects, as initial findings suggest the presence of vulnerable groups among non-medical students, such as those studying technology subjects [22, 27].

### Strengths and Limitations

This systematic review and meta-analysis provide a valuable estimation of the prevalence of depressive symptoms among higher education students in Germany. The study exhibits several strengths, including the comprehensive approach of searching three databases, the use of alerts for relevant publications until data extraction was completed, and a well-structured and exhaustive search strategy. Furthermore, the study’s adherence to PRISMA guidelines for systematic reviews, coupled with the quality appraisal conducted by two researchers, significantly bolsters the robustness of its findings.

However, it is essential to acknowledge the inherent limitations associated with this research. Utilizing self-report instruments is a valid method for assessing depressive symptoms. These instruments, despite targeting the same latent variable, exhibit variations in their conceptualization, including the application of distinct cut-off levels. This discrepancy served as the rationale for our subgroup analysis on the two out of 14 most frequently employed instruments. Interestingly, we found similar prevalence rates for the PHQ and BDI, even though one assesses moderate and the other mild depressive symptoms. However, our study has notable limitations due to strong over-representation of research with focus on students from medical subjects, the heterogeneity observed among the results, and the variability in measurement tools among the included studies. While subgroup analyses were conducted to explore potential sources of heterogeneity, a more in-depth assessment through meta-regression was not performed, leaving the underlying causes of this heterogeneity not fully elucidated. Accordingly, it is not possible, for example, to determine why there were differences in prevalence rates before and during the COVID-19 pandemic. In addition, these differences need to be interpreted with caution, because they were based on different studies with different sample characteristics and procedures.

Regarding the quality assessment of the articles, we employed a standardized tool to reduce potential biases. Most of the studies featured adequate sample sizes, sampling methods, and measurement of assessing depressive symptoms. Studies with a poor quality (<5 out of 9 possible points) accounted for a relatively small proportion (4,495/75,107 participants = 5.98%) of the overall sample, so that an overall low risk of bias can be assumed. However, there was variability in the quality of reporting in prevalence studies. In this regard, we can fully support the recommendation for the incorporation of checklists like STROBE [107] to ensure comprehensive and consistent reporting practices also in the context of future epidemiological research in Germany.

## Conclusion

In conclusion, this systematic review with meta-analysis sheds light on the significant burden of depressive symptoms among higher education students in Germany. Our findings indicate that up to 30% (in certain subgroups) are affected by depressive symptoms, a prevalence higher than in the general population calls for urgent attention and intervention. Importantly, our analysis reveals variations in pooled prevalence rates across different subgroups, highlighting the need for targeted mental health support tailored to specific student demographics such as female sex. Ongoing monitoring and support should be provided to ensure the wellbeing and academic success of higher education students. Universities should consider revising study conditions and implementing support programs that address the mental health needs of their student populations. This may include providing accessible counselling services and fostering a supportive learning environment.

Furthermore, the impact of the COVID-19 pandemic on mental wellbeing of higher education students cannot be underestimated. Our study underscores that depressive symptom prevalence rates were notably higher during the pandemic compared with pre-pandemic times. This reflects the unique challenges and stressors faced by students during this unprecedented period. Against the background of persistent crises, it is essential to delve deeper into the specific factors contributing to the increased prevalence of depressive symptoms among higher education students. Longitudinal studies that track students’ mental health over an extended period can provide valuable insights into the lasting effects of the pandemic on their wellbeing.
