# Supplementary material for: Depressive Symptoms Among Higher Education Students in Germany—A Systematic Review and Meta-Analysis
Source: Public Health Rev. 2024 Jun 10;45:1606983. doi: 10.3389/phrs.2024.1606983 (PMC11228579; doi:10.3389/phrs.2024.1606983)
Supplement: Supplementary file 1 [file DataSheet1.docx]

Supplementary File 1: Pooled prevalence rates of depressive symptoms among subgroups (Depressive symptoms among higher education students in Germany – a systematic review and meta-analysis, Berlin, Germany, 2022-2023)

| **Subgroup** | **Pooled prevalence rate (%)** | **95 % Confidence Interval (%)** | | **Heterogeneity (I², %)** |
| --- | --- | --- | --- | --- |
|  |  | **lower** | **upper** |  |
| PHQ scale | 24.8 | 19.5 | 30.1 | 100 |
| BDI scale | 22.4 | 17.4 | 27.3 | 97 |
| Female students | 29.0 | 21.4 | 36.5 | 99 |
| Male students | 23.1 | 16.5 | 29.6 | 98 |
| Medical students | 13.2 | 9.9 | 16.4 | 97 |
| First-year students | 11.0 | 7.7 | 14.3 | 93 |
| Before the COVID-19 pandemic | 18.0 | 14.7 | 21.2 | 99 |
| During the COVID-19 pandemic | 30.6 | 22.1 | 39.1 | 100 |

PHQ = Patient Health Questionnaire; BDI = Beck Depression Inventory; COVID-19 = Corona Virus Disease 2019

Supplementary File 2: Stratified pooled prevalence rates of depressive symptoms among subgroups (before the pandemic) (Depressive symptoms among higher education students in Germany – a systematic review and meta-analysis, Berlin, Germany, 2022-2023)

| **Subgroup analysis** | **Pooled prevalence rate (%)** | **95 % Confidence Interval (%)** | | **Heterogeneity (I², %)** |
| --- | --- | --- | --- | --- |
|  |  | **lower** | **upper** |  |
| BDI scale | 22.4 | 17.4 | 27.3 | 97 |
| PHQ scale | 18.6 | 13.5 | 23.8 | 99 |
| Female students | 25.7 | 18.8 | 32.5 | 99 |
| Male students | 20.1 | 14.2 | 26.1 | 97 |
| Medical students | 13.2 | 9.4 | 17.0 | 98 |

Supplementary File 3: Stratified pooled prevalence rates of depressive symptoms among subgroups (during the pandemic) (Depressive symptoms among higher education students in Germany – a systematic review and meta-analysis, Berlin, Germany, 2022-2023)

| **Subgroup analysis** | **Pooled prevalence rate (%)** | **95 % Confidence Interval (%)** | | **Heterogeneity (I², %)** |
| --- | --- | --- | --- | --- |
|  |  | **lower** | **upper** |  |
| PHQ scale | 33.7 | 25.1 | 42.2 | 99 |
| Medical students | 13.3 | 7.6 | 19.0 | 93 |
|  |  |  |  |  |
